# Automatic Kidney Image Segmentation During Robot-Assisted Partial Nephrectomy Using a Deep Learning Model Based on a Multiannotator Dataset: Model Development and Validation Study

**DOI:** 10.2196/82540

**Published:** 2026-09-02

**Authors:** Gaëlle Margue, Kilian Chandelon, Maxime Pattou, Alice Pitout, Abderrahmane Khaddad, Federico Rubat-Baleuri, Julie Desternes, Laura Richert, Nicolas Bourdel, Adrien Bartoli, Jean-Christophe Bernhard

**Affiliations:** 1 Department of Urology Bordeaux University Hospital Bordeaux France; 2 I.CaRe, Bordeaux Institute of Oncology French National Institute of Health and Medical Research Unit 1312 Bordeaux France; 3 Surgical Augmented Reality Clermont-Ferrand France; 4 French National Centre for Scientific Research and Université Clermont Auvergne Joint Research Unit 6602 Endoscopy and Computer Vision Research Group, Institut Pascal Clermont-Ferrand France; 5 Research in Clinical Epidemiology and Public Health, European Clinical Trials Platform and Development–French Clinical Research Infrastructure Network Bordeaux University Hospital, Bordeaux Population Health Research Center Unit 1219, Clinical Investigation Center–Clinical Epidemiology Unit 1401 University Bordeaux, French National Institute of Health and Medical Research, Institut Bergonié Bordeaux France; 6 Department of Obstetrics and Gynecology Clermont-Ferrand University Hospital Clermont-Ferrand France; 7 Department of Clinical Research and Innovation Clermont-Ferrand University Hospital Clermont-Ferrand France

**Keywords:** robotic partial nephrectomy, augmented reality, deep learning, kidney segmentation, image annotation, surgical guidance, convolutional neural networks, medical imaging, interannotator variability

## Abstract

**Background:**

Augmented reality (AR) has emerged as a promising tool to enhance surgical precision during robot-assisted partial nephrectomy (RAPN), particularly by enabling the intraoperative overlay of 3D anatomical models. However, real-time AR implementation requires robust image segmentation of anatomical structures, such as the kidney, which remains technically challenging in dynamic laparoscopic environments.

**Objective:**

This study aimed to develop and validate a large, annotated image dataset to train deep learning models for automated segmentation of the renal parenchyma during RAPN, as a prerequisite for real-time AR guidance.

**Methods:**

We conducted a single-center, observational image annotation study using prospectively collected surgical videos from 131 RAPN procedures performed between 2022 and 2024. Patients had localized renal tumors, with 11 presenting with multifocal disease (160 tumors in total). A total of 48,000 frames were extracted based on image sharpness, diversity, and the exclusion of artifacts. A subset of 454 images was annotated by 9 contributors (surgeons, engineers, and nonexperts) after structured training. Interannotator agreement was assessed using the Dice similarity coefficient (DSC) and sensitivity against an expert reference. A convolutional neural network (AlbuNet-34) was trained using 12,546 annotated images and evaluated on a validation set of 3137 images. Model performance was analyzed overall and across surgical phases.

**Results:**

Annotators achieved high agreement, with median DSC values ranging from 0.91 to 0.95 and sensitivity consistently more than 0.89. The deep learning model achieved a mean DSC of 0.75 (SD 0.23) and a sensitivity of 0.71 (SD 0.24) on the validation set. Segmentation accuracy varied significantly across surgical phases, with lower performance observed during tumor resection and tumor bed reconstruction due to increased visual complexity.

**Conclusions:**

This study demonstrates the feasibility of automated renal parenchyma segmentation using deep learning in real-world intraoperative settings. Although current performance remains below that of expert-level annotations, the creation of a large, annotated dataset and the implementation of a structured multiannotator workflow represent key milestones toward reliable AR-assisted surgery. Ongoing refinements in annotation quality, dataset diversity, and neural network optimization are expected to enhance future real-time AR applications in urology.

## Introduction

Partial nephrectomy (PN) is the gold standard for the management of localized kidney tumors, allowing preservation of renal function while maintaining excellent oncological outcomes [1,2]. The introduction of minimally invasive techniques and robotic assistance has further improved perioperative outcomes by reducing blood loss, complications, and hospital stays [3,4]. However, robot-assisted PN (RAPN) remains technically demanding, particularly for complex tumors, for which precise identification of resection planes is essential to minimize ischemic injury and achieve complete tumor excision [5,6].

To enhance spatial understanding, high-resolution contrast-enhanced computed tomography (CT) enables the creation of patient-specific 3D virtual models [7-9]. Integrating these models intraoperatively, as in 3D image-guided RAPN (3D-IGRAPN) [10], improves surgical navigation. Augmented reality (AR) extends this approach by overlaying digital information directly onto the surgical field, supporting real-time localization of anatomical structures [11,12]. Unlike virtual reality (VR), which immerses users in a simulated environment, AR enhances the operative scene through image registration and tracking [13,14].

**Figure 1:**
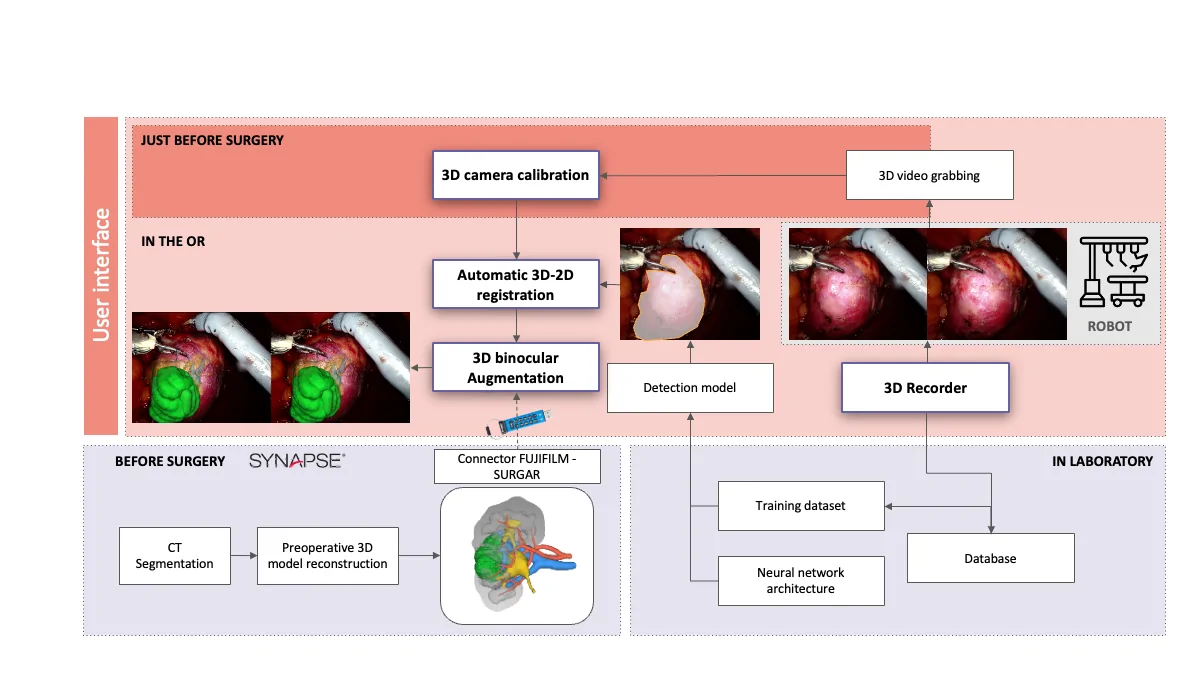
Schematic representation of the augmented reality guidance process during robot-assisted partial nephrectomy. CT: computed tomography; OR: operating room.

Surface-based registration facilitates alignment between preoperative 3D models and intraoperative images without requiring fiducial markers. This process relies on stereoscopic vision, in which 3D laparoscopic cameras triangulate corresponding points to reconstruct surface geometry. However, accurate registration depends on the prior recognition of the renal surface in intraoperative frames. Advances in computer vision and deep learning have made this possible by enabling automatic image segmentation and tracking of anatomical structures in real time [15]. Consequently, developing a robust AR system for RAPN requires training neural networks on large, diverse, and well-annotated datasets that capture the variability of intraoperative scenes. Figure 1 illustrates the overall workflow of an AR-guided RAPN procedure, in which intraoperative kidney segmentation forms the cornerstone for aligning preoperative 3D models with laparoscopic views.

Despite major advances in preoperative 3D modeling, intraoperative AR integration remains limited by the absence of large, multiannotator datasets that enable reliable real-time segmentation.

This study aimed to develop and validate a deep learning segmentation system for RAPN based on a large, multiannotator dataset. Specifically, we describe dataset construction, assess interannotator variability, and evaluate the performance of an automatic model designed to enable real-time kidney segmentation for AR-guided surgery.

## Methods

### Study Design and Population

To train a neural network for accurately segmenting the renal parenchyma in intraoperative images, a high-quality dataset was constructed through the following steps: image collection, anonymization, preprocessing, annotation, and validation.

Between 2022 and 2024, surgical videos of RAPN performed with the da Vinci surgical system (Intuitive Surgical) were prospectively collected at an expert center (Bordeaux University Hospital, France). The inclusion criteria were as follows: (1) patients diagnosed with localized kidney tumors, either solitary or multiple, with varying complexity, location, and size and (2) patients eligible for RAPN who did not object to the collection of their data for the study. Videos were recorded using the MVR Pro system (MediCapture), ensuring that high-resolution video streams (1280×1024 pixels) were maintained.

### Video Acquisition and Frame Extraction

Image extraction from the video footage was automated based on a predefined set of selection criteria to guarantee optimal quality and diversity. Image sharpness was assessed using Canny edge detection [16], which is based on the principle that sharp images exhibit a high density of well-defined contours. The sharpness score was computed as the ratio of detected edge pixels to the total image size, filtering out blurry or low-detail frames. A temporal spacing constraint was implemented to minimize redundancy by restricting frame extraction. This involved ensuring that frames were extracted only if the time interval between consecutive frames was >5 seconds. To achieve frame diversity, a Marr-Hildreth image hashing [17] algorithm was used. This technique computed the perceptual similarity between frames, discarding those with high redundancy based on a predefined Hamming distance threshold (set at 200). Extracted images were carefully reviewed to exclude artifacts and were subsequently annotated with metadata, including tumor location, size, and the RENAL (radius, exophytic/endophytic, nearness, anterior, location) nephrometry score [18]. The surgical procedures were divided into 5 critical phases: kidney release, tumor preparation, hilar dissection, tumor resection, and tumor bed reconstruction, each phase chosen for its relevance to AR-guided surgical improvements. All 131 RAPN procedures contributed usable images for each of the 5 predefined surgical phases (kidney release, tumor preparation, hilar dissection, tumor resection, and tumor bed reconstruction). Thus, every case was represented across all phases, ensuring a consistent phase-based distribution of annotated frames for training, validation, and testing.

### Annotation Workflow and Quality Control

To achieve high-quality annotation, diverse annotator groups were defined: 2 junior urologists (regularly assisting in RAPN procedures), 2 physicians from other specialties (medical professionals without expertise in RAPN), 2 medical engineers (with technical backgrounds in the segmentation process), 2 external annotators (individuals without surgical or anatomical expertise), and 1 expert surgeon (professor of urology with extensive experience in performing RAPN). All annotators received uniform structured training, leveraging a detailed guidebook and the Supervisely software (Supervisely OÜ), initially focusing on basic annotation principles before advancing to the nuances of renal surgery images. Annotators were required to demonstrate proficiency by achieving scores of at least 80% in general training and at least 90% in renal-specific tasks, ensuring consistency and accuracy.

**Figure 2:**
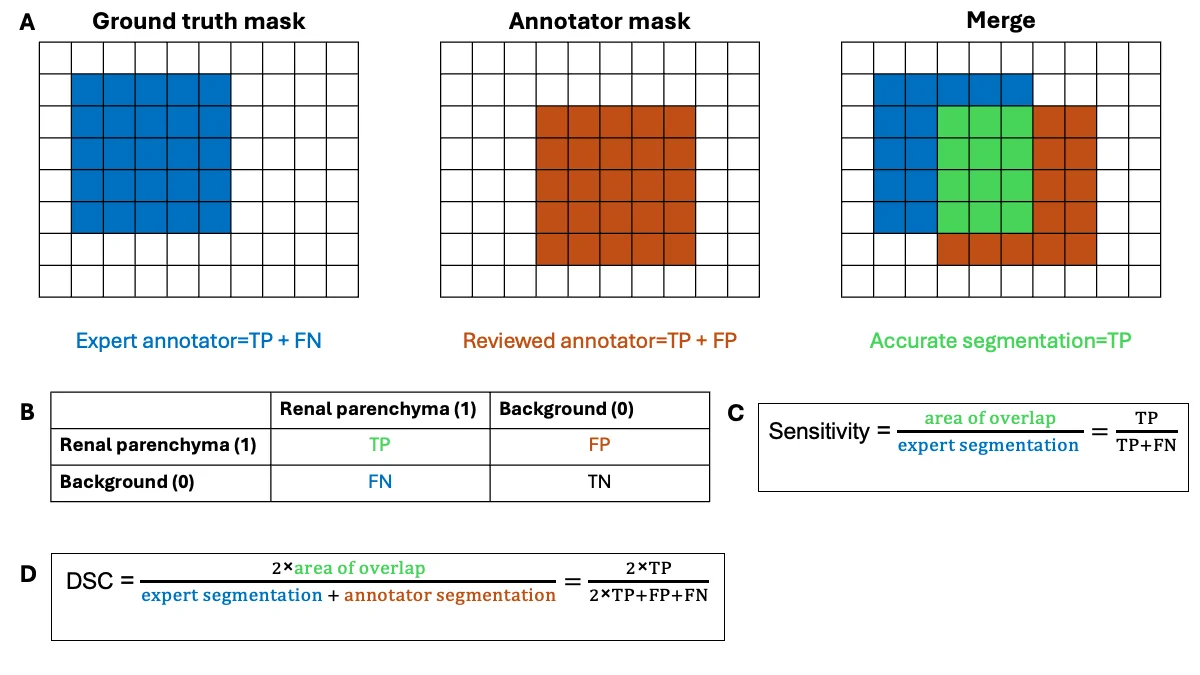
Segmentation metrics derived from a confusion matrix for renal parenchyma segmentation. (A) Example of a confusion matrix; (B) table defining true positive (TP), false positive (FP), false negative (FN), and true negative (TN); (C) equation for sensitivity; and (D) equations for the Dice similarity coefficient (DSC).

The reliability of annotated images was evaluated using sensitivity and the Dice similarity coefficient (DSC) [19] (Figure 2), compared to the ground truth provided by the expert surgeon. The DSC ranges from 0 to 1, with 1 indicating perfect agreement. Although no predefined threshold was set for an acceptable DSC in this study, values more than 0.8 are commonly considered indicative of good interannotator agreement.

Consistency across annotators was ensured through an evaluative dataset featuring 454 images categorized into 3 complexity groups: simple, intermediate, and complex. Simple cases included images with an easily identifiable renal surface (97/454, 21.4%). Intermediate complexity was defined by the presence of significant fat on the renal surface (78/454, 17.2%); blood or clots (66/454, 14.5%); or instruments, compresses, clips, or loops in front of the renal surface (98/454, 21.6%). Complex cases involved the presence of at least 2 of these factors (115/454, 25.3%). The structural similarity index measure (SSIM) was used to confirm diversity, focusing on luminance, contrast, and structural features. SSIM scores, ranging from 0% to 100%, increase with greater similarity. The mean SSIM score for our dataset was 35.9%, indicating sufficient diversity to test the segmentation process while maintaining structural integrity and consistency. Heat maps were generated to visually represent areas of consensus and discrepancies across annotators.

Annotations from external contributors underwent statistical analysis to gauge accuracy before inclusion in the neural network dataset. For quality control, 9 surgeries were randomly selected to ensure balanced representation of surgical phases and annotator groups. Each segmentation was compared to expert reference masks to quantify labelling accuracy. A segmentation was considered correct when the predicted contour overlapped more than 70% with the expert-defined renal surface. A 30% error threshold was defined, based on prior internal validation experience, to trigger reannotation of uncertain or inconsistent cases. This relatively permissive threshold was selected as a pragmatic quality control criterion to identify clearly incorrect segmentations in complex intraoperative conditions, rather than to define optimal segmentation performance. This process allowed the quantification of overall annotation reliability and the identification of recurrent labelling errors before inclusion in the dataset. We calculated 95% CIs for the proportion of correctly annotated images.

To further assess annotation reliability, intraannotator variability analyses were conducted on a subset of the evaluation dataset. A total of 100 images were selected, including 20 images from each predefined complexity group. These images were annotated twice by the expert annotator.

### Model Development and Training

**Figure 3:**
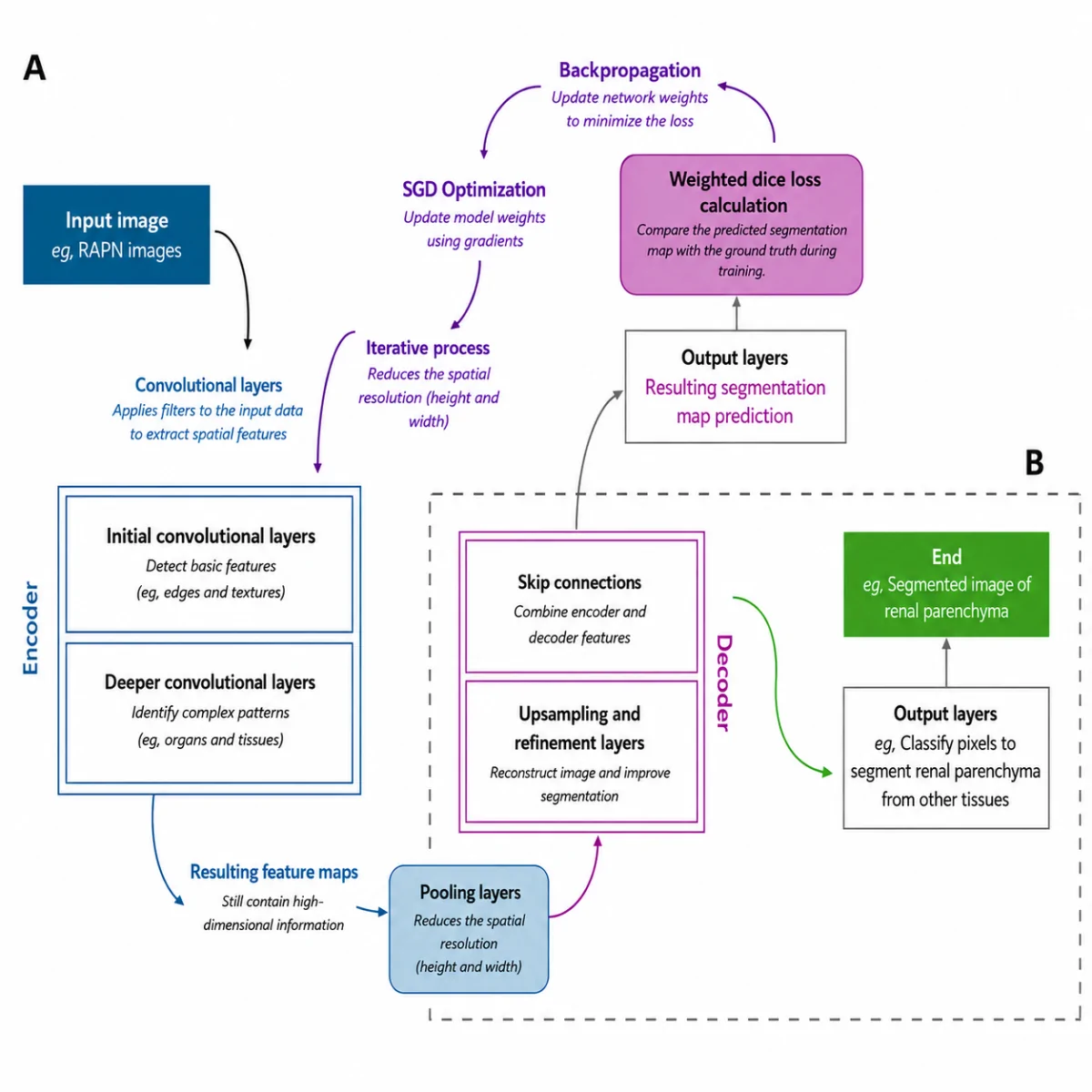
Schematic representation of the convolutional neural network architecture (AlbuNet-34), illustrating (A) the training phase and (B) the inference phase. SGD: stochastic gradient descent.

AlbuNet-34 [20], a U-Net architecture–based [21] deep convolutional neural network (CNN), was implemented to automate renal parenchyma segmentation (Figure 3). The choice of the AlbuNet-34 architecture was guided by preliminary comparative experiments conducted during the development phase of the AR pipeline, prior to the present study. Conventional U-Net–based architectures and U-Net variants with different pretrained encoders (including ResNet-34, MobileNet-v2, and EfficientNet-B3) were evaluated under identical training conditions to assess convergence stability, segmentation accuracy, and computational efficiency in complex intraoperative scenes. AlbuNet-34 provided the most robust tradeoff between segmentation performance and computational cost, which motivated its selection for the present study. These comparisons focused on CNN-based architectures compatible with real-time intraoperative constraints. Although several architectures were explored during preliminary experiments, these comparisons were not conducted as part of a formal benchmarking framework and are therefore not reported quantitatively.

The annotated dataset was divided into a training set of 12,546 images and a validation set of 3137 images, with all images standardized to a resolution of 512×512 pixels. All frames from a given RAPN surgery were assigned to the same data subset to prevent data leakage. Consequently, no case contributed images to more than 1 partition. To address the strong class imbalance inherent to intraoperative images, a weighted Dice loss was applied to emphasize the minority class (renal parenchyma). Preliminary experiments without class weighting resulted in reduced sensitivity for renal parenchyma segmentation, which was detrimental to downstream surface tracking. The adopted loss function provided improved training stability and higher recall for the target organ.

The training process spanned 30 epochs, with a batch size of 8 to balance computational efficiency and convergence speed. Stochastic gradient descent (SGD) was used for optimization, starting with an initial learning rate of 0.001. To enhance convergence, an automatic learning rate scheduler reduced the learning rate by a factor of 0.1 every 5 epochs, preventing overshooting as training advanced. No explicit data augmentation strategy was applied during training. Preliminary augmentation experiments were conducted using standard techniques, including geometric transformations (rotations), photometric adjustments (brightness and contrast variations), and noise injection. However, these approaches did not yield consistent improvements in segmentation performance. Given the intrinsic variability of intraoperative images, including illumination changes, occlusions, bleeding, and tissue deformation, these strategies were not retained.

Model performance was rigorously assessed through sensitivity and DSC metrics, comparing its segmentation outputs to those of expert annotations.

### Statistical Analysis

Segmentation performance was assessed using multiple complementary metrics: the DSC and the intersection over union (IoU) to quantify spatial overlap, sensitivity (recall) and specificity to evaluate pixel-wise detection performance, and precision (positive predictive value [PPV]) to characterize false-positive control.

For each image, all metrics were computed for each annotator. Results are summarized as both the mean (SD) and median (IQR) at the per-image level, as distributions were nonnormal (Shapiro-Wilk test: *P*<.001).

Interannotator agreement was analyzed by grouping annotators into four expertise groups: (1) senior surgeons (“Physicians”), (2) junior urologists, (3) medical engineers, and (4) external annotators without prior urological training.

To assess phase-specific performance, each annotated frame was assigned to a predefined surgical phase (kidney release, tumor preparation, hilar dissection, tumor resection, and tumor bed reconstruction). All 454 evaluated frames were mapped to one of these phases (n=215, 47.4% kidney release; n=91, 20% tumor preparation; n=50, 11% hilar dissection; n=42, 9.3% tumor resection; and n=56, 12.3% tumor bed reconstruction).

Statistical comparisons between groups were performed using the Kruskal-Wallis test.

Exact *P* values and effect sizes are reported. Statistical significance was defined as *P*<.05.

Analyses and plots were generated using R (version 4.4.2; R Foundation for Statistical Computing).

### Ethical Considerations

This study was approved under the UroCCR protocol 122 (ClinicalTrials NCT03293563), with data processing authorized by the CNIL (DR-2013-206). All patients provided written informed consent for the use of their surgical data for research purposes. Patient confidentiality was preserved through rigorous pseudonymization techniques implemented using the Scientific Python Development Environment (Spyder; version 5) and the *FFmpeg* library. This involved removing identifiable information and applying pseudonymization, with the resulting encrypted dataset stored securely with restricted access.

## Results

### Study Population and Dataset

To establish a comprehensive dataset for training an automated renal parenchyma recognition system, we gathered 131 RAPN surgical videos over 3 years. All surgeries provided usable annotated images for each of the 5 predefined surgical phases, resulting in a balanced dataset of 48,000 frames covering the full intraoperative workflow. A separate subset of 454 manually annotated frames was used exclusively for interannotator variability analysis and was not included in the training or validation datasets. Patient demographics are summarized in Table S1 in Multimedia Appendix 1. Among the included patients, 11 presented with multiple tumors, resulting in a total of 160 lesions. Detailed tumor characteristics (eg, location, size, proximity to critical structures, and RENAL nephrometry scores) are reported in Table S2 in Multimedia Appendix 2. A small subset of videos lacked patient identifiers and could therefore be used only for technical analysis, without linkage to individual clinical data.

### Annotation Performance

**Figure 4:**
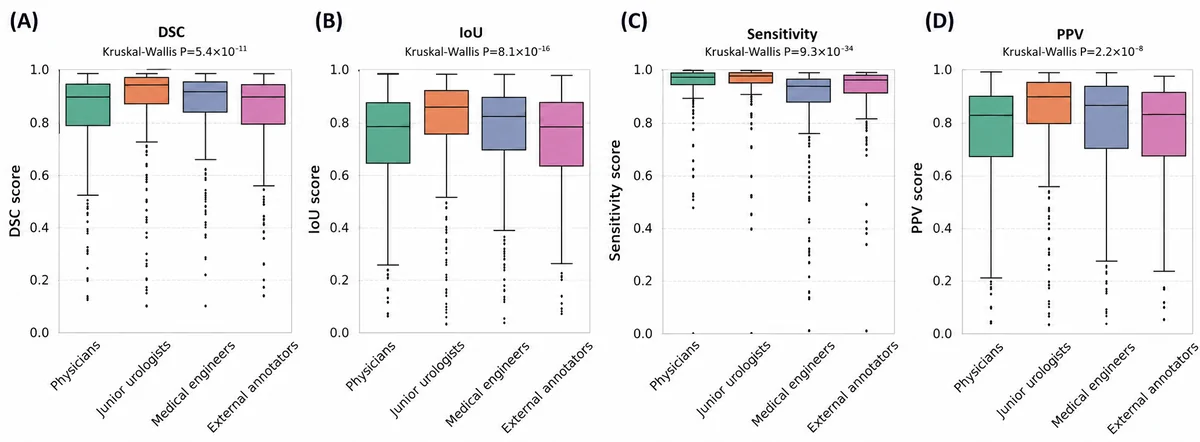
Boxplots of Dice similarity coefficient (DSC), intersection over union (IoU), sensitivity, and positive predictive value (PPV) across annotator groups. Boxes show median (IQR), and whiskers indicate 5th to 95th percentiles. Kruskal-Wallis *P*<.001 for all metrics.

Segmentation performance was evaluated across 4 annotator groups (n=454 manually annotated images): senior surgeons (“Physicians”), junior urologists in training, medical engineers, and external annotators without prior urological experience. Across all groups, DSC, IoU, sensitivity, and PPV varied significantly (Kruskal-Wallis *P*<.001 for all comparisons; Table 1; Figure 4).

**Table 1:** Percentage of annotations achieving Dice similarity coefficient (DSC), intersection over union (IoU), sensitivity, and positive predictive value (PPV) scores >0.8, stratified by annotator.

Annotators	Annotations with DSC >0.8, % (95% CI)	Annotations with IoU >0.8, % (95% CI)	Annotations with sensitivity >0.8, % (95% CI)	Annotations with PPV >0.8, % (95% CI)
Physician 1	73.1 (68.8-77.1)	70.8 (66.5-75.1)	93.8 (91.2-95.9)	81.2 (77.1-85.3)
Physician 2	72.3 (67.9-76.3)	69.7 (65.4-74.0)	92.3 (89.4-94.6)	80.4 (76.2-84.6)
Junior urologist 1	83.9 (80.2-87.2)	81.5 (77.6-85.3)	93.8 (91.2-95.9)	89.3 (86.0-92.6)
Junior urologist 2	77.1 (72.9-80.9)	75.0 (70.7-79.3)	93.4 (90.7-95.5)	85.7 (81.8-89.6)
Medical engineer 1	77.5 (73.4-81.3)	74.2 (70.0-78.4)	86.8 (83.4-89.6)	84.1 (80.2-87.9)
Medical engineer 2	77.3 (73.1-81.1)	73.5 (69.2-77.8)	85.7 (82.2-88.6)	83.2 (78.8-87.5)
External annotator 1	72.3 (67.9-76.3)	70.1 (65.8-74.4)	82.2 (78.4-85.4)	79.6 (75.1-84.0)
External annotator 2	70.0 (65.6-74.2)	67.9 (63.5-72.3)	92.9 (90.2-95.0)	80.2 (75.9-84.6)

Junior urologists achieved the highest overall performance, with a median DSC of 0.95 (IQR 0.87-0.98), IoU of 0.91 (IQR 0.78-0.96), PPV of 0.93 (IQR 0.79-0.98), and sensitivity of 0.98 (IQR 0.96-0.99). Physicians achieved a median DSC of 0.91 (IQR 0.77-0.96), IoU of 0.83 (IQR 0.64-0.92), PPV of 0.86 (IQR 0.63-0.96), and sensitivity of 0.97 (IQR 0.95-0.99). Medical engineers achieved a median DSC of 0.92 (IQR 0.83-0.97), IoU of 0.86 (IQR 0.71-0.93), PPV of 0.91 (IQR 0.68-0.99), and sensitivity of 0.95 (IQR 0.88-0.97), while external annotators achieved a median DSC of 0.91 (IQR 0.78-0.96), IoU of 0.83 (IQR 0.64-0.92), PPV of 0.86 (IQR 0.64-0.95), and sensitivity of 0.97 (IQR 0.91-0.98; Table 1). Specificity remained uniformly high across all groups (≥0.97), confirming a low false-positive rate.

**Figure 5:**
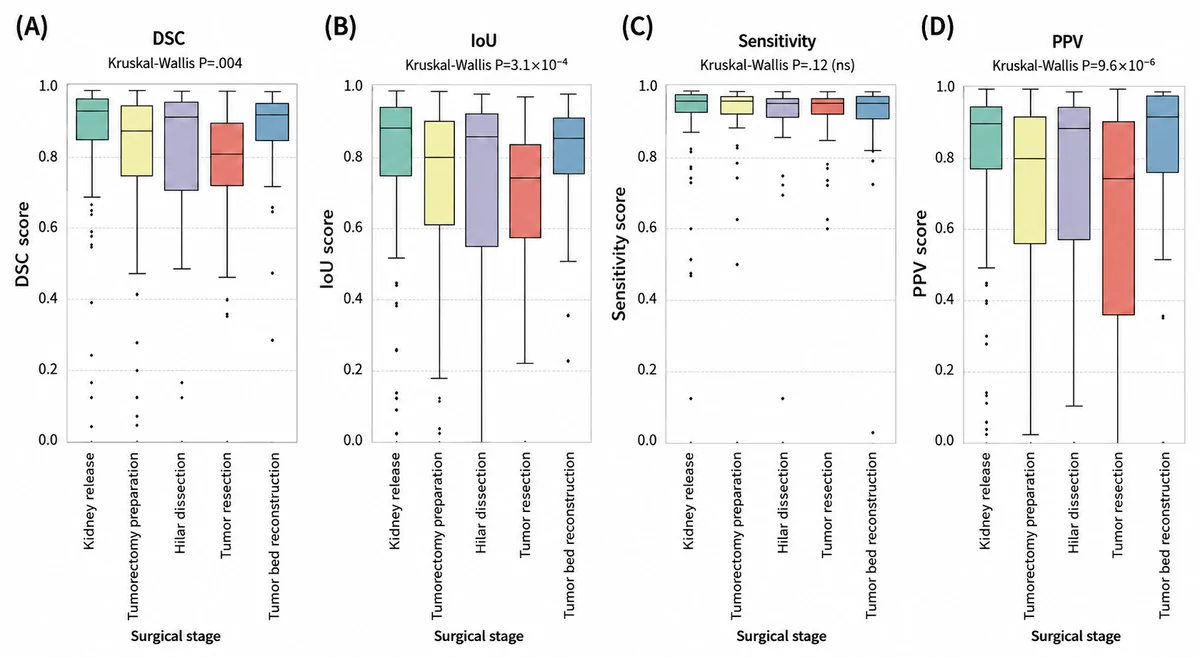
Boxplots of the Dice similarity coefficient (DSC), intersection over union (IoU), sensitivity, and positive predictive value (PPV) stratified by surgical phase (kidney release, tumorectomy preparation, hilar dissection, tumor resection, and tumor bed reconstruction). Boxes show median (IQR), and whiskers indicate 5th to 95th percentiles. Kruskal-Wallis *P*=.004 for DSC, and *P*=.12 for sensitivity.

Between-group differences were significant for all metrics (Kruskal-Wallis *P*<.001 for DSC, *P*<.001 for IoU, *P*<.001 for sensitivity, and *P*<.001 for PPV). Overall, annotation accuracy was high (median DSC>0.90, IoU>0.80, and PPV>0.85), and trained junior urologists achieved the best balance between overlap and pixel-wise detection. Heat maps (Figure 5) provided visual overlays of segmentation overlap and discrepancies among the 9 annotators. Detailed per-annotator metrics (median and IQR for DSC, IoU, sensitivity, and PPV) are provided in Table S3 in Multimedia Appendix 3.

**Figure 6:**
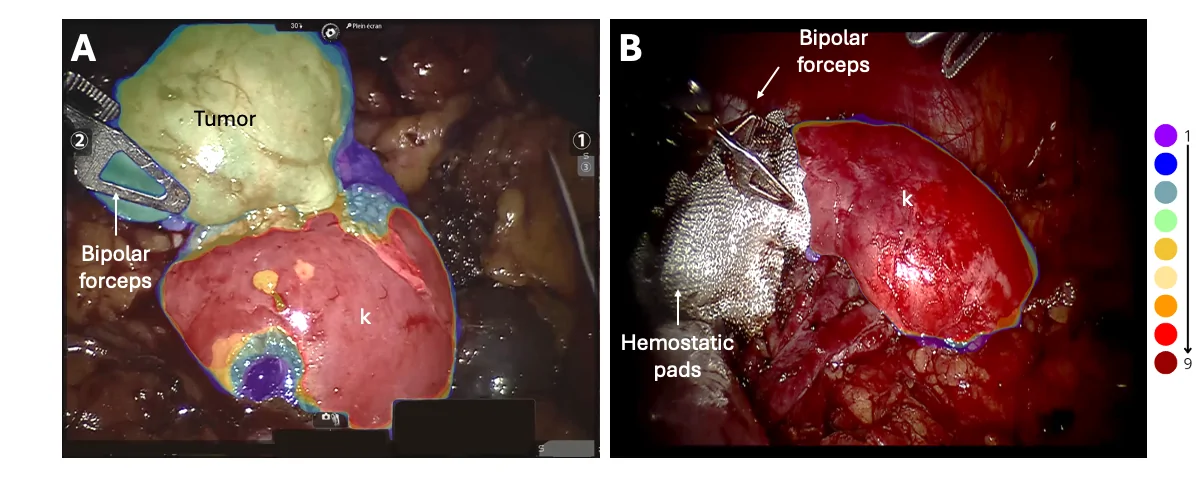
Overlay of manual renal parenchyma segmentations during 2 surgical phases: (A) renal dissection and (B) tumor bed reconstruction, showing interannotator variability. Dark red indicates agreement among all 9 annotators, and purple indicates annotation by only 1 annotator.

Phase-based evaluation revealed differences in interannotator agreement across the 5 surgical phases (Figure 6). Median Dice scores relative to the expert reference were 0.94 (IQR 0.87-0.97) during kidney release, 0.90 (IQR 0.75-0.96) during tumor preparation, 0.92 (IQR 0.79-0.96) during hilar dissection, 0.90 (IQR 0.73-0.96) during tumor resection, and 0.95 (IQR 0.86-0.97) during tumor bed reconstruction.

Phase-based differences were statistically significant for most metrics (Kruskal-Wallis *P*=.004 for DSC, *P*<.001 for IoU, and *P*<.001 for PPV), whereas sensitivity remained stable across phases (*P*=.12), suggesting consistent pixel-wise detection even during technically demanding steps. Lower overlap metrics during the resection and preparation phases were consistent with occlusions, blood, and instrument interference, whereas the dissection and reconstruction phases maintained higher consistency. Full phase-specific results for each annotator group are summarized in Table S4 in Multimedia Appendix 4.

Intra-annotator variability analysis demonstrated high repeatability of expert annotations, with a median DSC of 0.94 (IQR 0.91-0.97) and a median IoU of 0.89 (IQR 0.84-0.94), depending on image complexity.

### Annotation Efficiency and Quality Control

Annotation time varied markedly between groups (Kruskal-Wallis *P*<.001). External annotators required the most time (mean 4 minutes 24 seconds, SD 3 minutes 21 seconds per image) and medical engineers required the least time (mean 40, SD 23 seconds per image). High SDs (Table S5 in Multimedia Appendix 5) reflect substantial interparticipant variability, emphasizing the need for standardized annotation protocols.

External annotations were audited to ensure reliability. Despite their limited background, they achieved an average DSC of >0.81, contributing to the 48,000-image dataset. A targeted reevaluation of 9 surgeries (5459 images; Table 2) showed narrow 95% CIs for cases with ≥90% correct segmentation, supporting annotation precision. The global correct segmentation rate was 68.9% (SD 17.4%), with most errors arising from boundary misclassification and oversegmentation or undersegmentation. No correlation was found between the success rate and tumor characteristics.

**Table 2:** Assessment of external annotators’ segmentation quality by expert review for 9 robot-assisted partial nephrectomy (RAPN) procedures^a^.

Surgery (RAPN)	Segmentations judged correct by the expert reviewer, n/N (%; 95% CI)
RAPN 1	147/294 (50; 44-56)
RAPN 2	357/608 (58.72; 55-63)
RAPN 3	274/281 (97.51; 95-99)
RAPN 4	336/646 (52.01; 48-56)
RAPN 5	910/1005 (90.55; 89-92)
RAPN 6	530/759 (69.83; 66-73)
RAPN 7	437/618 (70.71; 67-74)
RAPN 8	449/864 (51.97; 49-55)
RAPN 9	301/384 (78.3-9; 74-82)

^a^Mean number of images per surgery 607 (SD 251); mean number of segmentations judged correct by the expert reviewer, 51.92% (216/416).

### Model Performance

**Figure 7:**
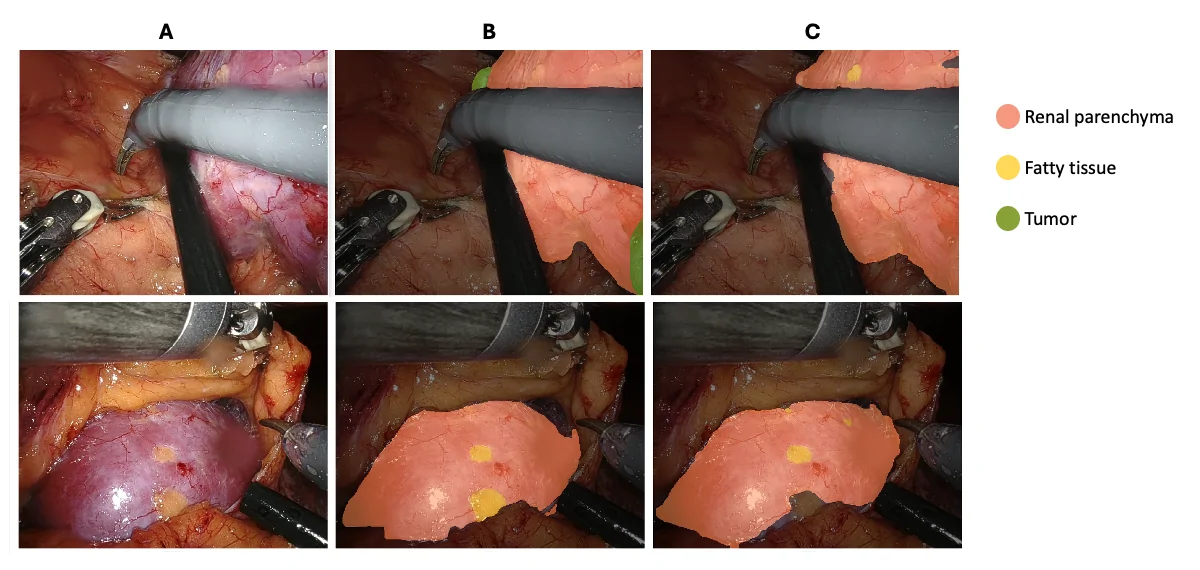
Visual comparison of manual and automatic renal parenchyma segmentations. (A) Examples of intraoperative images, (B) corresponding expert manual segmentations, and (C) automatic segmentations generated by the AlbuNet-34 model.

Using the manually annotated dataset, the AlbuNet-34 CNN achieved a mean DSC of 0.75 (SD 0.23) and a mean sensitivity of 0.71 (SD 0.24) against the expert ground truth. Figure 7 illustrates these comparisons across images of varying complexity. The model was trained on 12,546 images and validated on 3137 images, requiring approximately 10 hours and 24 minutes of computation.

Although these results confirm the feasibility of automatic renal parenchyma segmentation, model performance remains below that of expert-level segmentation, particularly during complex surgical phases, such as tumor resection and tumor preparation, where occlusions, bleeding, and instrument interference affect visual clarity.

## Discussion

### Principal Findings

In this study, we developed and validated a large multiannotator dataset to enable automatic kidney segmentation during RAPN. This work represents one of the first feasibility demonstrations of intraoperative segmentation for AR guidance, bridging preoperative 3D virtual modeling with near–real-time surgical vision. Importantly, the objective of this study was not to establish a state-of-the-art segmentation benchmark but to assess the feasibility and robustness of intraoperative kidney segmentation as a functional component of an AR pipeline. Given the growing evidence supporting the benefits of 3D virtual models in RAPN [10,22], our study specifically addresses the remaining gap: the intraoperative application of AR to achieve dynamic anatomical guidance.

Integrating AR into RAPN represents a shift from traditional imaging, overcoming the limitations of 2D modalities, such as ultrasound, CT, and magnetic resonance imaging (MRI), which fall short in dynamic surgical settings. AR offers real-time, 3D visualization, enhancing spatial understanding and decision-making [23]. This innovation is particularly critical for complex tasks, such as endophytic tumor localization and the preservation of critical structures, showing AR’s potential to improve surgical precision, safety, and patient outcomes. Beyond preoperative 3D-IGRAPN, which has already demonstrated clinical benefits [10,22], our dataset and segmentation model address this intraoperative gap by providing quantitative validation of their feasibility.

Accurate real-time segmentation is crucial for the seamless overlay and alignment of digital models onto the surgical field. Surface-based registration plays a key role in enhancing the localization of renal surfaces and enabling image fusion without the need for physical markers. This capability is achieved through advanced stereoscopic vision and 3D camera technology, which facilitates precise spatial triangulation and the creation of an intraoperative 3D model derived from the renal surface topography. This intraoperative model is aligned with the virtual 3D model generated from preoperative CT scans. Surgical guidance using AR benefits from the combination of detection and tracking of the renal parenchyma, and the successful implementation of these processes relies on the initial recognition of the renal surfaces within intraoperative images using a trained neural network.

The development of a reliable parenchyma segmentation system relies on extensive datasets that are critical for accurate near–real-time organ segmentation. Our work highlights the time-consuming nature of manual segmentation, corroborating the findings of De Backer et al [24], who reported that 1248 hours were required to segment 15,100 frames in a study on real-time instrument delineation. Similarly, our time analysis highlighted substantial variability in the time annotators spent per image, with high SDs across groups (Table S5 in Multimedia Appendix 5). This variability likely contributed to the absence of statistically significant differences in segmentation performance between annotator groups. These findings reflect the labor-intensive and cognitively demanding nature of the segmentation task, particularly given the large number of images involved. Together, these findings reinforce the importance of optimizing annotation workflows and potentially developing semiautomated tools to alleviate annotator burden.

Using nonexpert annotations effectively expands the dataset, alleviating the burden on medical professionals. Interannotator variability remains a challenge, with DSC scores often less than 0.70 for complex organs [25,26]. Additionally, recent studies underscore reduced identifiability for retroperitoneal organs, such as the kidneys, compared with intraperitoneal organs [27]. Despite these challenges, in our study, we obtained DSC scores higher than those reported in the existing literature [25,26], suggesting that trained nonexperts can contribute meaningfully to data annotation. However, the observed variability highlights the need for careful supervision and training to ensure annotation reliability. Importantly, quality control analysis confirmed that expert oversight remains essential, with approximately 69% of segmentations judged to be correct according to predefined quality control criteria based on expert overlap assessment.

To optimize nonexpert contributions, structured training and advanced annotation tools are critical. Providing clear guidelines and feedback loops through expert review enhances annotation accuracy, provides valuable learning opportunities for nonexperts, and improves dataset quality. Expanding the image source beyond a single expert center will increase dataset variability, making the dataset more representative of different clinical conditions and enhancing the AR system’s utility and reliability in real-world surgical environments.

Phase-specific analysis revealed that segmentation performance varied significantly across surgical steps. Kidney release and reconstruction phases showed the highest agreement, while tumor resection and tumor preparation phases were more challenging due to occlusions, bleeding, and tissue deformation. These results highlight that the segmentation output is most reliable during the exposure and reconstruction phases, whereas selective activation during resection may optimize usability and reduce cognitive load.

Real-time performance remains challenging because rapid data processing is crucial for continuous updates during surgery. Preliminary integration experiments conducted during the development of the AR framework suggest that CNN-based renal parenchyma segmentation is compatible with near–real-time deployment on graphics processing unit (GPU)–based systems. However, these experiments were performed outside the scope of the present study and under different experimental conditions; therefore, a formal evaluation of latency and frame rate within an end-to-end AR pipeline is required to confirm real-time feasibility and clinical usability. Addressing latency [28], reducing equipment bulk, and improving user interfaces are essential steps toward broader adoption and practical application in surgical environments. Precise initial model positioning on organs is crucial for accurate alignment and tumor localization but remains a challenge due to difficult-to-identify anatomical landmarks on radiological and laparoscopic images [29]. Kidney appearance varies among patients, which can affect landmark visibility. Currently, manual registration is required; however, automating this process would streamline surgical workflows, allowing surgeons to focus entirely on the procedure. These real-time and integration constraints currently favor lightweight CNN-based architectures over more computationally demanding model families.

Our rigid registration framework highlights the need for models that accommodate soft tissue deformation. Addressing this limitation can significantly improve real-time modeling of organ dynamics, thereby refining the precision of AR tools. Our findings revealed phase-dependent variability in segmentation accuracy, highlighting the dynamic challenges of maintaining consistent AR performance throughout RAPN. Segmentation performance was highest during kidney release and reconstruction, whereas tumor resection and tumor bed reconstruction were more challenging because of increased visual complexity, including bleeding, instrument occlusion, and rapid anatomical changes. In this context, similar to ultrasound, AR guidance may be most effective when applied on demand at critical moments rather than continuously, thereby enhancing visualization when needed while minimizing unnecessary cognitive or technical burden. These phase-specific limitations also emphasize the importance of refining annotator training protocols and annotation tools for visually complex stages, as well as implementing ongoing quality control to improve dataset consistency and robustness.

More broadly, few robust studies have successfully segmented kidney structures from laparoscopic videos, underscoring the complexity of such imagery and the lack of standardized benchmarks in this domain. In this context, architectural choices were guided by robustness and computational efficiency under intraoperative constraints rather than by exhaustive benchmarking against multiple model families. A more systematic comparison of modern architectures, including lightweight CNNs and transformer-based models, would be valuable for further optimizing segmentation performance and should be addressed in future studies. The relatively low DSC score of our neural network, compared to those reported in CT- or MRI-based studies [30], arises from the unique challenges of laparoscopic images, such as lower resolution, variable lighting, and motion artifacts. These challenges present opportunities for innovation in improving segmentation accuracy. Although our model currently underperforms compared to human annotators, we anticipate that the performance of AlbuNet-34 will improve with time. Enhanced by data from the UroCCR network and our guidebook, which highlights annotator challenges, continuous training and additional data are expected to elevate the model’s performance toward human-level accuracy. The AlbuNet-34 architecture was selected based on preliminary comparative experiments conducted during the development phase of the AR framework, in which several U-Net–based variants were evaluated under identical conditions. These comparisons aimed to identify a robust and computationally efficient solution compatible with intraoperative deployment, rather than to optimize performance on generic segmentation benchmarks.

The development of AR systems for RAPN exemplifies interdisciplinary collaboration, merging expertise in medicine, engineering, and computer science to enhance clinical viability and technological sophistication. Real-life trials are crucial to validate the effectiveness of AR systems and ensure that they translate into tangible improvements in renal oncology surgery, culminating in better patient outcomes.

This study has several limitations. First, its single-center design and the absence of external validation may limit the generalizability of our findings. Second, although the reference standard was defined by a single expert annotator, intraannotator variability analysis demonstrated high repeatability (IoU>89% and DSC>93%). However, this analysis does not fully capture interexpert variability, and the lack of multiexpert consensus annotations may introduce systematic bias. Third, model evaluation was performed on a validation set without an independent held-out test set, which may lead to optimistic performance estimates. Fourth, segmentation performance varied across surgical phases, reflecting differences in visual complexity, and real-time feasibility was not formally assessed, particularly regarding latency and frame rate. Finally, although architectural choices were guided by robustness and computational efficiency, systematic benchmarking and ablation studies were not exhaustively performed, and some redundancy may persist in the dataset despite frame selection strategies. Future work should address these limitations through multicenter validation, multiexpert annotation, independent test datasets, and more comprehensive model comparisons.

### Conclusions

This study presents the initial steps toward developing an AR system for RAPN, emphasizing interdisciplinary collaboration to enhance surgical precision. It validates the feasibility of intraoperative kidney segmentation based on a large multiannotator dataset and outlines the next step toward multicenter, real-time validation. Despite challenges related to segmentation variability and real-time processing, leveraging deep learning together with structured training for nonexpert annotators demonstrates promising potential. Future efforts will focus on model optimization, latency measurement, and clinical validation.

## Data Availability

The datasets generated and analyzed during the current study are not publicly available because of patient confidentiality and institutional restrictions but are available from the corresponding author on reasonable request and subject to ethics approval.

## Appendix

Multimedia Appendix 1 Clinical characteristics of patients with robot-assisted partial nephrectomy from our dataset.

Multimedia Appendix 2 Characteristics of kidney tumors in patients with robot-assisted partial nephrectomy from the dataset (n=160 tumors in 131 patients).

Multimedia Appendix 3 Per-annotator segmentation performance.

Multimedia Appendix 4 Comparison of segmentation performance metrics (median [IQR]) between annotator groups across surgical phases.

Multimedia Appendix 5

## Abbreviations

3D-IGRAPN: 3D image-guided robot-assisted partial nephrectomy
AR: augmented reality
CT: computed tomography
DSC: Dice similarity coefficient
GPU: graphics processing unit
IoU: intersection over union
MRI: magnetic resonance imaging
PN: partial nephrectomy
PPV: positive predictive value
RAPN: robot-assisted partial nephrectomy
RENAL: radius, exophytic/endophytic, nearness, anterior, location
SGD: stochastic gradient descent
SSIM: structural similarity index measure
VR: virtual reality
